# Examining the Impact of Microglia on Ischemic Stroke With an Emphasis on the Metabolism of Immune Cells

**DOI:** 10.1111/cns.70229

**Published:** 2025-02-13

**Authors:** Jing Lv, Yang Jiao, Xinya Zhao, Xin Kong, Yanwei Chen, Lu Li, Xuyang Chen, Xufeng Tao, Deshi Dong

**Affiliations:** ^1^ Department of Pharmacy First Affiliated Hospital of Dalian Medical University Dalian China; ^2^ College of Pharmacy Dalian Medical University Dalian China; ^3^ Department of Neurology First Affiliated Hospital of Dalian Medical University Dalian China; ^4^ Dalian Innovation Institute of Stem Cell and Precision Medicine Dalian China

**Keywords:** immunometabolism, ischemic stroke, microglia, neuroinflammation

## Abstract

**Background:**

Ischemic stroke, a major cause of disability and the second leading cause of death, poses a significant public health challenge. Post‐stroke inflammation can harm the blood–brain barrier and worsen neurological deficits, which are key factors in neuronal damage in patients with ischemic stroke. Microglia are crucial in the central nervous system, involved in inflammation, neuronal damage, and repair after cerebral ischemia. While cellular immune metabolism has been widely studied, its role in ischamic stroke remains unclear.

**Aim:**

This review aims to examine how inflammation affects the phenotypic characteristics of immune cells after ischemic stroke and to explore the effects of the immune metabolic microenvironment on the phenotypic profiles and functions of microglia in ischemic stroke.

**Method:**

The review refers to the available literature in PubMed, searching for critical terms related to Ischemic stroke, neuroinflammation, microglia, and immunometabolism.

**Result:**

In this review, we found that during stroke progression, microglia can dynamically switch between pro‐inflammatory and anti‐inflammatory phenotypes. Microglial glycometabolism includes oxidative phosphorylation and glycolysis, and lipid metabolism involves lipid synthesis and breakdown. Modulating the production of inflammatory mediator precursors can induce an anti‐inflammatory phenotype in microglia.

**Conclusion:**

Thus, studying microglial metabolic pathways and their products may offer new insights for ischemic stroke treatment.

## Graphical abstract

After ischemic stroke, resting microglia transform into pro‐inflammatory or anti‐inflammatory microglia under different conditions, respectively. Moreover, microglia respond to the environment by flexibly reprogramming intracellular metabolic pathways, thereby altering immune function, which mainly includes glucose metabolism, lipid metabolism, and amino acid metabolism.

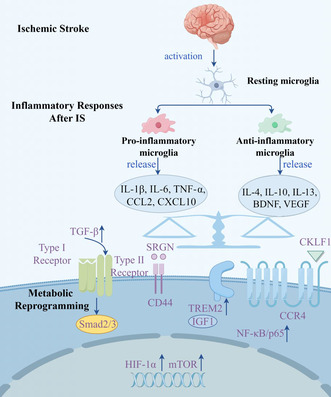

## 1 Introduction

Stroke impacts more than 25% of adults aged 25 years and older, with more than 12.2 million individuals experiencing a stroke annually. As the second most common cause of death and a significant contributor to disability worldwide, stroke affects millions of individuals each year [1]. Stroke is typically categorized as either ischemic or hemorrhagic, with the majority of cases classified as ischemic [2]. In IS, ischemia and hypoxia lead to energy impairment, ion balance disruption, and pH level imbalances. These various dysfunctions have the potential to trigger neuropathological alterations within the brain, such as inflammation [3], problems with cell energy production [4], excitotoxicity [5], damage to the blood–brain barrier [6], and cell death [7]. Without restoration of blood flow, the cells in the area surrounding the blocked blood vessel die, which induces the formation of the central ischemic region.

Existing therapies for IS focus on achieving prompt reperfusion through intravenous thrombolysis and/or endovascular thrombectomy, which have demonstrated success in decreasing disability [8]. The crucial aspect of treating IS is the swift reopening of the obstructed blood vessels to preserve the ischemic penumbra. The sole medication approved by the FDA for the treatment of acute IS is recombinant tissue plasminogen activator (r‐tPA). The therapeutic time window for alteplase and tenecteplase is within 4.5 h after the onset of symptoms. For patients who meet the criteria for treatment (under the age of 80), excluding contraindications, the benefits of treatment are time‐dependent and should be initiated as soon as possible [9, 10, 11]. However, only half of the patients who receive this drug fully recover [12]. Despite these advancements, the application of these therapeutic modalities remains encumbered by numerous challenges, including the narrow therapeutic time window for thrombolysis/thrombectomy, the peril of hemorrhagic transformation, and the scarcity of highly efficacious and safe drugs on the market. These challenges significantly impede the acute management and long‐term rehabilitation of IS patients.

Multiple preclinical and clinical studies have indicated that secondary brain injury occurs in addition to primary craniocerebral injury and is exacerbated by additional lesions, which lead to further harm to the brain [13]. Moreover, the inflammation that occurs after IS plays a crucial role in the development of secondary damage. The inflammatory process involves the stimulation of the brain's resident immune cells, including microglia and border‐associated macrophages, as well as the infiltration of peripheral leukocytes into the perihematomal ischemic region [14, 15]. The excessive production of proinflammatory cytokines by stimulated microglia/macrophages and peripheral leukocytes is triggered, which in turn causes cerebral edema, disrupts the blood–brain barrier, and leads to neuronal cell death [16]. Following cerebral ischemia, microglia become activated and undergo complex processes that include cytokine secretion, chemotaxis, phagocytosis, and proliferation. Microglia migrate to the affected ischemic zone to clear away dead cells and produce neurotrophic factors that support the survival of neurons [17]. Additionally, microglia release inflammatory cytokines and cytotoxic compounds that may worsen ischemic damage [18, 19, 20, 21, 22]. As a result, anti‐inflammatory treatment could be a viable approach for helping individuals recover from stroke. Microglia play crucial roles in preserving the balance within the CNS and are vital for brain progression and the management of various disorders.

In the 1920s, the Nobel Prize recipient Otto Warburg first discovered differences in the metabolic patterns between normal tissues and cancer cells, which laid the foundation for subsequent research on the metabolism of immune cells. Immune cells respond to their surroundings by adaptively changing internal metabolic pathways, which in turn modify immune function in a phenomenon known as immunometabolism [23, 24]. The immune reaction following a stroke has recently become a novel focal point of the therapeutic approach for IS. Microglia, myeloid cells found in the brain, are key players in the innate immune system and function in constant monitoring for immune responses. Nevertheless, the effects of metabolic reprogramming on microglia, neuroinflammation, and ultimately, brain function in the CNS, remain unclear.

In recent years, the significant role of microglial immune metabolism in IS has garnered considerable attention, which has led to remarkable advancements in related research. This area of study is pivotal in deepening our comprehension of the underlying pathogenesis of IS. Furthermore, a greater understanding of this topic will provide a robust scientific foundation for the development of innovative and effective treatment strategies, which are highly important for improving the prognosis and the quality of life of individuals who have experienced a stroke. This study delves into the effects of the immune metabolic microenvironment on the microglial phenotype and its influence on IS, highlighting three key aspects: carbohydrate, lipid, and amino acid metabolism. This study aims to provide new research perspectives for the treatment of ischemic stroke by studying the metabolic pathways and products of microglia.

## 2 Inflammatory Responses After IS

**FIGURE 1:**
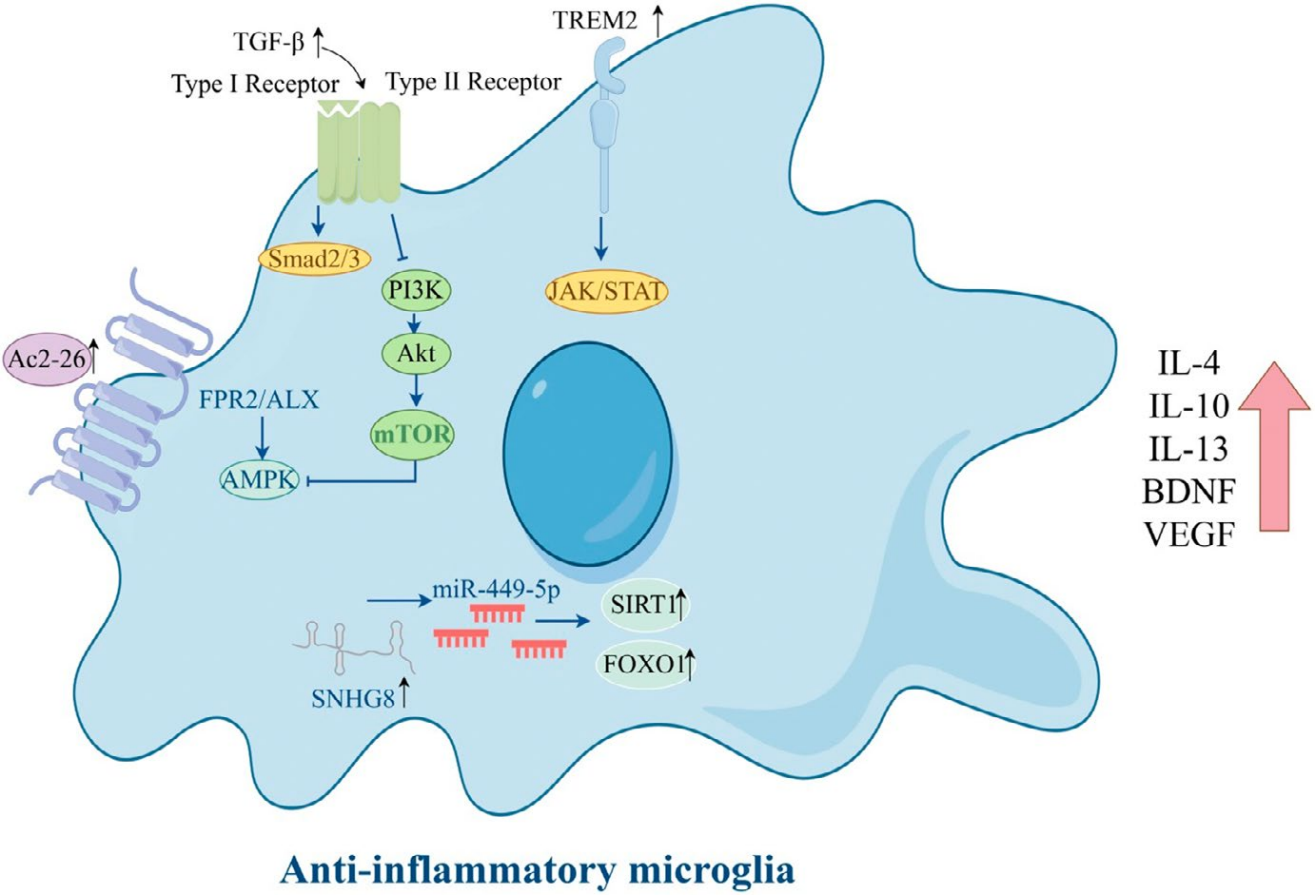
Schematic diagram of anti‐inflammatory phenotype polarization of microglia. (By Figdraw).

Activation of microglia was initially classified into two functional categories: classical activation into a proinflammatory M1 phenotype and an alternative activation promoting repair and immunosuppression, termed M2. Here, we summarize the diagram of microglial polarization towards an anti‐inflammatory phenotype (Figure 1). While this is now considered an oversimplification of the complex variations of microglia inflammatory responses that are possible, it is clear that microglia respond to changes in brain homeostasis by modifying their phenotype, with each challenge driving a unique reactive state. In the proinflammatory state, different inflammatory agents and toxic substances are released. In damaged neural tissue, the expression of cytokines TNF‐α, IL‐1β, and inducible nitric oxide synthase (iNOS) by microglia is enhanced, exacerbating cellular damage, whereas the neuroprotective phenotype can engulf cell pieces and release substances that promote nerve growth and reduce inflammation (Table 1). Interleukin‐10 (IL‐10), arginase‐1 (Arg‐1), and brain‐derived neurotrophic factor cooperate to facilitate the healing of injured tissue. Given the ability of the two activated states of microglia to transition between each other, targeted drug interventions aimed at enhancing neuroprotective effects by converting microglia to the M2‐like (anti‐inflammatory) phenotype could offer therapeutic advantages for acute IS [25, 26, 27].

**TABLE 1:** Microglia in post‐stroke neuroinflammation.

Inflammatory cytokine	Outcome	References
IL‐1β	Induces microglia proinflammatory gene expression	[18]
IL‐6	Induces microglia proinflammatory gene expression	[18]
TNF‐α	Induces microglia proinflammatory gene expression	[18, 19]
IL‐10	Induce anti‐inflammatory gene expression in microglia	[26]
IL‐4	Induce anti‐inflammatory gene expression in microglia	[27]
HMGB1	Stimulate the release of inflammatory cytokines and the expression of iNOS	[20, 21, 22]

### 2.1 Phenotype of Microglia After IS

In 1919, with the breakthrough in specific silver staining techniques, Río‐Hortega precisely described the morphological features of the third largest cell population in the brain, named this population microglia. Río‐Hortega carefully noted that microglia experience notable changes in shape following brain damage, establishing a crucial basis for future studies [28].

In the initial phase of acute IS, microglia display unique diversity. Single‐cell RNA sequencing analysis revealed three homeostatic groups, as well as five reactive groups which predominantly appeared post‐IS. The Mic_M1L1 and Mic_M1L2 groups presented characteristics of M1‐like polarization due to increased inflammatory gene expression following IS. Three distinct cell clusters, Mic_np1, Mic_np2, and Mic_np3, exhibit low levels of inflammation and are distinguished by elevated levels of Arhgap45, Rgs10, and Pkm expression, respectively [29]. The phenotypic changes in microglia are not confined to only the M1/M2 polarization state; rather, they can exhibit more complex and diverse phenotypes under different physiological and pathological conditions. Additional investigations are needed to explore the mechanisms underlying microglial phenotypic alterations to develop successful treatment strategies for neurological disorders.

Microglia, the primary immune cells in the CNS, are activated in the context of cerebral ischemia/reperfusion injury (CIRI), which leads to the production of numerous proinflammatory mediators that contribute to secondary damage [30]. On the contrary, microglia can provide further neuroprotection by secreting a range of anti‐inflammatory substances and growth factors that are crucial for sustained neuroprotection and recovery from ischemic events [31]. Research indicates that microglial polarization is influenced by various factors, including nuclear factor kappa‐B (NF‐κB) [32, 33, 34, 35, 36]. Typically, NF‐κB, which is present in the cytoplasm and bound to the inhibitory protein IκB, is suppressed and forms a dimeric structure. The nuclear localization signal of NF‐κB is obscured by IκB, which renders it inactive. In the context of cerebral ischemic damage, cells are activated, leading to the degradation of IκB proteins through phosphorylation. Through exposure to a nuclear localization signal, NF‐κB is activated and translocates to the nucleus, where it regulates transcription to increase the synthesis and expression of inflammatory mediators, ultimately worsening brain injury [37, 38]. CIRI is decreased by mesencephalic astrocyte‐derived neurotrophic factor (MANF), which regulates the anti‐inflammatory phenotype polarization of microglia via the A20/NF‐κB pathway [32]. Monocyte locomotion inhibitory factor (MLIF) induces the transition of microglia to the anti‐inflammatory phenotype. The NF‐κB pathway plays a role in the effects of MLIF by targeting eukaryotic elongation factor 1A1e (EF1A1) [36]. Erythropoietin‐producing human hepatocellular receptor A4 (EphA4) upregulation exacerbates brain damage by promoting proinflammatory phenotype polarization of microglia through NF‐κB signaling after cerebral ischemia [34]. NF‐κB‐regulated chemokine‐like factor 1 (CKLF1) upregulation in neurons is a key mechanism in promoting proinflammatory phenotype of microglia in the ischemic penumbra [35]. Salvianolic acid D, identified as a polyphenol within the plant *Salvia miltiorrhiza* Bunge, alleviates CIRI by suppressing the cytoplasmic translocation and release of high mobility group box 1 (HMGB1)‐triggered NF‐κB activation to inhibit inflammatory response (Table 1); [39]. Additionally, a variety of natural substances, including triptolide, β‐patchoulene, ginkgetin, tanshinone IIA, breviscapine, diosgenin, icariin, and berberine, have been shown to protect the brain from ischemic injury. This protective effect is mediated by their ability to inhibit the NF‐κB signaling pathway [40, 41, 42, 43, 44].

Inhibiting proinflammatory phenotype polarization and promoting anti‐inflammatory phenotype polarization of microglia is a suggested therapeutic strategy to reduce neuroinflammation and enhance neuroprotection following CIRI. Microglia proinflammatory phenotype polarization leads to poor prognosis in IS patients and is associated with systemic inflammation. The activation of solute carrier 15A3 (SLC15A3) by p65 and hypoxia‐inducible factor 1 alpha (HIF1α) leads to microglial polarization towards the proinflammatory phenotype [45]. By inducing microglia to become proinflammatory phenotype polarized via the AMP‐activated protein kinase (AMPK) signaling pathway, long noncoding RNA nuclear paraspeckle assembly transcript 1 (NEAT1) suppresses the angiogenic activity of cerebral artery endothelial cells [46]. Patients who experience acute ischemic stroke (AIS) have increased numbers of cerebral microglia that express high levels of Netrin‐1 and Unc‐5 netrin receptor A (UNC5a) [47]. In vitro, Netrin‐1 facilitates anti‐inflammatory phenotype polarization of microglia via UNC5a. Swell1 may also be involved in the regulation of the WNK1‐SPAK/OSR1‐NKCC1 signaling pathway, which helps promote microglial anti‐inflammatory phenotype polarization [48]. During CIRI, the JAK2/STAT3 signaling pathway is involved in regulating microglia and macrophage polarization towards the anti‐inflammatory phenotype [49]. Protein arginine methyltransferase 8 (PRMT8) promotes the anti‐inflammatory phenotype polarization of microglia and reduces neuronal apoptosis to improve CIRI by increasing Lin28a expression [50]. Ac2‐26, an ANXA1 mimetic peptide, protects against CIRI by shifting microglial/macrophage polarization from the proinflammatory phenotype to the anti‐inflammatory phenotype, possibly by interacting with the FPR2/ALX receptor and activating the AMPK–mTOR pathway [51]. Triggering receptor expressed on myeloid cells 2 (TREM2) is involved in regulating the shift of microglia towards the anti‐inflammatory phenotype via a range of signaling pathways including the TGF‐β/Smad2/3 pathway [52], the JAK/STAT pathway [53], and the PI3K/Akt pathway [54]. Additionally, lncRNA SNHG8 [55] and Wip1 [56] can regulate microglial activation in CNS disorders (Figure 1).

### 2.2 Interactions of Microglia With Neurons

After IS, neurons are the primary cells affected and are also engaged in multiple regulatory functions that are closely associated with microglia. In summary, in both pathological and physiological states, neurons have the ability to regulate the activation of microglia through signaling mechanisms known as “On” and “Off” [57]. Following ischemia, the activation of microglia is first initiated by neuronal death [58]. Furthermore, microglia play various roles in the regulation of neurons. One example is the ingestion of nerve cells [59]. Following a cerebral ischemic event, activated microglia engulf dead or deteriorating neurons, which aids in the recovery process [60]. Recent research has indicated that in the penumbra area, the complement pathway directs the phagocytosis of stressed but recoverable neurons by microglia, which suggests promising targets for future therapeutic studies.

### 2.3 Interactions of Microglia With Other Glial Cells

#### 2.3.1 Interactions of Microglia With Astrocytes

Astrocytes are the most abundant type of glial cells in the mammalian CNS. After IS, astrocytes become activated and transition from resting state to the reactive state. Reactive astrocytes upregulate numerous genes, increase the volume of the cytoskeleton, enhance the expression of glial fibrillary acidic protein (GFAP) and immune reactivity, and form glial scars [61]. Neuroinflammation and stroke produce two distinct types of reactive astrocytes, A1 proinflammatory and A2 anti‐inflammatory [62]. Microglia and astrocytes are key regulators of neuroinflammation, and when the brain is damaged, they play their respective roles through complex interactions.

Reactive astrocytes release chemokines and cytokines (like MCP‐1 and IL‐10), modulate the activity of microglia, promote the rapid migration of microglia to the damaged area, and facilitate the transformation of microglia into a neuroprotective phenotype [63]. Whether in vivo or in vitro, the activation of microglia can induce the formation of A1 reactive astrocytes by secreting three cytokines: IL‐1α, TNF‐α, and C1q (complement component 1, subcomponent q), leading to a cascade of neuroinflammation [64]. In IS, PRDX6 is primarily expressed in astrocytes. PRDX6‐iPLA2 plays a key role in ROS production induced by astrocytes and the activation of microglia, which are regulated by the activation of Nox2 and Drp1‐dependent mitochondrial fission pathways. Microglia protect astrocytes from ischemic injury by secreting interleukin‐1 receptor antagonist (IL‐1RA) to inhibit astrocyte CXCL1 and reduce neutrophil‐mediated microthrombosis, thereby decreasing ischemic damage. Blocking CXCL1 with specific antibodies or delivering exogenous rIL‐1RA may provide therapeutic possibilities to improve the prognosis of IS [65]. Studies have found that astrocyte‐derived extracellular vesicles can inhibit the activation of the NF‐κB/NLRP3 signaling pathway, suppress the inflammatory response of N9 microglia treated with oxygen and glucose deprivation (OGD) in vitro, and reduce OGD‐induced N9 microglia pyroptosis. In vivo, they inhibit the expression of pro‐inflammatory cytokines after middle cerebral artery occlusion (MCAO) in rats, reduce cerebral infarction in rats, and thus improve neurological function [66]. Current research on the crosstalk between microglia and astrocytes mainly focuses on other CNS diseases. Although these studies are of great significance, it is necessary to further investigate to determine the complex connections in IS.

#### 2.3.2 Interactions of Microglia With Oligodendrocytes

Researchers have reported that oligodendrocytes are particularly susceptible to ischemia, which typically leads to the breakdown of the myelin structure following a stroke [67]. This damage is strongly linked to decreased function in certain CNS conditions. Impairment in the differentiation of proliferating oligodendrocyte progenitor cells (OPCs) into oligodendrocytes leads to a lack of remyelination, which impedes neurological recovery poststroke [67]. Studies indicate that the interplay of microglia and oligodendrocytes plays an essential role in remyelination following stroke. One study has shown that inflammatory substances released by activated microglia can hinder oligodendrocytes/OPCs [57]. This study also revealed that the transition of proinflammatory states to the neuroprotective phenotype is linked to the remyelination process. Anti‐inflammatory phenotype, which serves as protective cells poststroke, can promote oligodendrocyte maturation during the remyelination process, which is a crucial aspect of successful remyelination [68]. Microglia play a significant role not only in disease states but also in maintaining the balance of OPCs during CNS development [69]. Further studies are needed to investigate the precise mechanism by which microglia support oligodendrocytes at various poststroke stages.

## 3 Metabolic Response of Microglia After IS

### 3.1 Glycometabolism of Microglia

**FIGURE 2:**
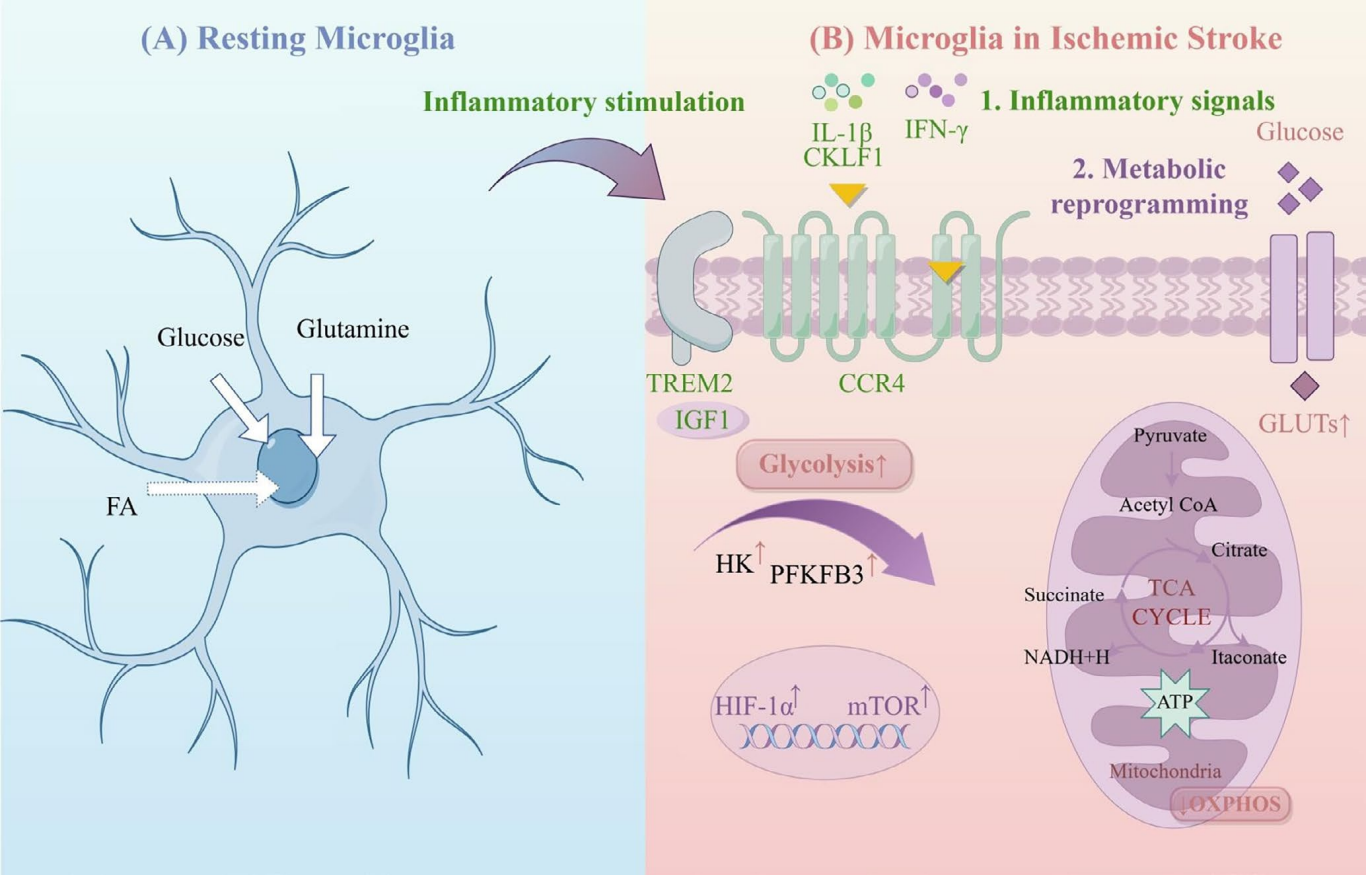
Metabolic Reprogramming of Microglia. (By Figdraw). (A) In a healthy brain, microglial cells exhibit metabolic flexibility, preferentially metabolizing glucose. Glutamine metabolism via glutaminolysis supports the energy demands of microglial cells. The utilization of fatty acids for ATP production remains controversial. (B) Following ischemic stroke, metabolic reprogramming occurs in microglial cells. Stimulated by various inflammatory signals, microglia reprogram their intracellular metabolic pathways. Reprogramming is characterized by increased glycolytic flux, accumulation of certain TCA metabolites, decreased mitochondrial respiration, and increased transcriptional control of HIF‐1α and mTOR. CCR4, C‐C Chemokine Receptor Type 4; CKLF1, Chemokine‐like factor 1, FA, fatty acid; GLUTs, Glucose transporters; HIF‐1α, hypoxia inducible factor‐1α; HK, hexokinase; IFN‐γ, interferon‐γ; IGF1, Insulin‐like Growth Factor 1; IL‐1β, interleukin‐1β; mTOR, mammalian target of rapamycin; OXPHOS, oxidative phosphorylation; PFKFB3, 6‐phosphofructo‐2‐kinase/fructose‐2,6‐biphosphatase 3; TCA, tricarboxylic acid.

The glycometabolism of microglia primarily involves the transformation between oxidative phosphorylation (OXPHOS) and glycolysis (Figure 2). In the acute phase of IS, due to the sharp decline in cerebral blood flow, along with the resulting ischemia and hypoxia in brain tissue, the glycometabolism of microglia transitions into the glycolysis pathway to meet the energy required for the synthesis of various proinflammatory factors under hypoxic conditions [70, 71, 72, 73, 74]. The pentose phosphate pathway is another main pathway of glycometabolism and is interchangeable with glycolysis, shares the G‐6‐P step, and presents a competitive inhibitory relationship. Its main product is a reduced coenzyme, which dominates the reduction reaction in the process of biosynthesis [75]. Excessive glycolysis in microglia can cause excessive accumulation of lactic acid in local tissues, which lowers the pH value and activates the pentose phosphate pathway to offset the excessive reactive oxygen species produced by glycolysis and maintain the redox balance of cells. Here, we summarize the potential mechanisms underlying microglial metabolic abnormalities in the pathogenesis of IS (Table 2).

**TABLE 2:** Potential mechanisms of abnormal metabolism in microglia in the pathogenesis of IS.

Abnormal metabolism	Potential metabolism leading to immune dysfunction	References
Glucose metabolism	Glycolysis↑; Lactic acid↑; Pyruvate↑; Succinic acid↑; Itaconic acid↑	[71, 72, 73, 74, 76, 77, 78]
	The Trem2‐IGF1 signaling pathway increases the level of OXPHOS in microglia	[79, 80]
	SRGN induces the activation of the NF‐kB p65 signaling pathway and enhances glycolysis in microglia	[81, 82, 83, 84, 85]
	CKLF1 activates the AMP‐AMPK–mTOR–HIF‐1α signaling pathway, increasing glycolysis in microglia	[86, 87]
	RvD1 enhances microglial glutamine uptake and stimulates glutaminolysis to support OXPHOS	[88, 89]
Lipid metabolism	Overexpression of TREM2 in microglia may alleviate neuroinflammation by modulating the TGF‐β1/Smad2/3 signaling pathway and regulating cholesterol synthesis	[52]
	SREBP1 promotes lipid synthesis and induces alternative activation in cells	[90]
Amino acid metabolism	Glutamate accumulates in large quantities in cerebral infarction lesions, producing neurotoxicity	[89, 91, 92]

TREM2 plays a crucial role similar to that of various phagocytic receptors, moving among the cytoplasm and membrane to regulate microglial phagocytic function and provide immune protection through anti‐inflammatory effects [93, 94]. Additionally, TREM2 influences the metabolic well‐being of microglia [79], particularly in maintaining homeostasis of glucose, insulin, cholesterol, HDL, and LDL [95, 96]. Research indicates that TREM2 dysfunction results in reduced cerebral blood flow and decreased glucose metabolism in the brain [80]. In ischemic brain tissue, an increased microglial subpopulation shows elevated levels of insulin‐like growth factor 1 (IGF1) and TREM2, along with increased OXPHOS activity [79]. IGF1, a 70‐amino acid polypeptide hormone [97], is known for its roles in development, proliferation, survival, antiaging, and neuroprotective effects across various diseases [98, 99]. IGF1 acts as a crucial downstream component of TREM2. The TREM2‐IGF1 signaling pathway serves as the foundation for microglial metabolism under cerebral ischemia–reperfusion conditions, where it promotes microglial proliferation and exerts neuroprotective effects by increasing the level of OXPHOS in microglia. Understanding the importance of activating the TREM2‐IGF1 signaling pathway in microglia is crucial for the treatment of IS.

Studies indicate a significant increase in microglial serglycin (SRGN) levels in the brain after an ischemic event [81]. The SRGN protein influences microglial activity by interacting with the CD44 receptor. SRGN worsens neuroinflammation following stroke and impedes recovery after IS by enhancing the proinflammatory response mediated by microglia. Furthermore, activation of the NF‐kB p65 signaling pathway and enhancement of glycolysis in microglia are induced by SRGN. Previous research has identified CD44 as a known receptor for SRGN, which demonstrates its involvement in the immune response [82, 83] and glucose/lipid metabolism [84, 85]. Targeting SRGN could offer new approaches to reduce brain injury after stroke.

Research has indicated that CKLF1 is significantly upregulated following cerebral ischemia [86]. The investigation revealed that CKLF1 triggers immediate microglial inflammation and shifts in energy processing from OXPHOS to glycolysis, which is dependent on the AMP‐AMPK–mTOR–HIF‐1α pathway [87]. Subsequently, activated microglia transition into a prolonged tolerant phase due to widespread irregularities in energy metabolism, which lead to decreased immune responses such as cytokine secretion and phagocytosis. The weakening of the immune response from microglia worsens the effects of stroke, but this effect could be improved by blocking or neutralizing CKLF1, which suggests a new direction for the treatment of IS.

Microglia need considerable energy to perform various tasks, such as engulfment and monitoring [100, 101]. Homeostatic microglia mainly use OXPHOS to generate ATP, whereas inflamed microglia rely on glycolysis [102, 103]. Resolvin D1 (RvD1) is a lipid mediator known for its strong anti‐inflammatory effects. Derived from docosahexaenoic acid (an omega‐3 polyunsaturated fatty acid) within the body [104], RvD1 enhances macrophage phagocytosis and reduces inflammation in experimental autoimmune neuritis [88]. Moreover, RvD1 enhances microglial glutamine uptake and stimulates glutaminolysis to support OXPHOS, increasing ATP production contingent on AMP‐AMPK activation, while also shifting energy metabolism from glycolysis to OXPHOS, which ensures an abundant energy supply for microglial phagocytosis [89].

Research has shown that reversing high levels of histone H3 lysine 27 acetylation (H3K27ac) inhibits the activity of overactive brain inflammation‐related genes [105]. The enzyme AMPK plays a crucial role in producing ATP through aerobic glycolysis [106, 107]. Increasing phosphoglycerate kinase 1 (PGK1) through the p300/H3K27ac pathway encourages microglia to become proinflammatory and inflamed during oxygen–glucose deprivation by controlling glycolysis [108].

Itaconic acid plays a crucial role as a key metabolite in immune metabolic reprogramming in glucose metabolism, which leads to significant impacts on immunity and host defense [76, 77]. Itaconate and its variants suppress tyrosine kinase protein 1, increase macrophage nuclear factor erythroid 2‐related factor 2 protein activity, reduce the expression of proinflammatory proteins, and influence the proinflammatory differentiation of microglia and macrophages poststroke. In IS, itaconate can inhibit the activation of adenosine‐activated protein kinases in microglia and macrophages, reduce intracellular lipid synthesis and accumulation, and alleviate inflammatory response‐induced injury [78, 109].

### 3.2 Lipid Metabolism in Microglia

The lipid metabolic pathway is an important part of microglial phenotypic regulation. Lipid synthesis metabolism generates the raw materials for inflammatory mediator precursors such as IL‐1β, leukotriene, and prostaglandin. The increased breakdown of lipids can lead to the conversion of microglia into an anti‐inflammatory state and increase the efficiency of lipid metabolism in microglia [110]. Following a stroke, CD11c+ microglia demonstrate an increased ability to engulf particles, elevated levels of genes related to lipid metabolism (Abca1, Abcg1, Apoe, Apoc1, and Lpl), and genes that support myelin, thereby accelerating the repair of white matter after an IS [110, 111].

Microglia depend on their lipid metabolism to fulfill their energy requirements during activation and when they execute effector tasks [112]. Under hypoxic conditions, cells react to stress signals by activating the transcription factor HIF‐1α. Treatment of the cells with cobalt chloride induces microglial activation through HIF‐1α. Sterol regulatory element binding protein 1 (SREBP1) plays a crucial role in controlling lipid production, whereas PPARα oversees the breakdown of fats and the absorption of fatty acids. Research has shown that when activated, microglia need continuous lipid production and fatty acid absorption, as shown by increased levels of SREBP1 and PPARα. By depleting antioxidant defense mechanisms, SREBP1 promotes lipid synthesis and enables alternative activation of cells [90]. The oxidative stress observed during microglial activation, characterized by elevated mtROS and iNOS gene expression, may result from weakened antioxidant defense mechanisms in the cells, which lead to the promotion of the phenotypic switch. Additionally, SREBP1‐mediated lipid synthesis also plays a role in regulating their phagocytic activity [90].

Microglia lacking TREM2 show a reduced ability to engulf particles and an increase in the storage of cholesteryl ester, which results in the formation of lipid droplets and an increase in the expression of perilipin‐2 (PLIN2) following hypoxia (Table 2). Silencing of TREM2 leads to an increase in lipid production (PLIN2, SOAT1) and a decrease in cholesterol elimination and lipid breakdown (LIPA, ApoE, ABCA1, NECH1, and NPC2), which further pushes microglia toward a proinflammatory phenotype and decreased TGF‐β1 levels in an IS model [52]. The overexpression of TREM2 in microglia may help alleviate postischemic neuroinflammation and neuronal cell death, at least partially, through the TGF‐β1/Smad2/3 signaling pathway and cholesterol production (Figure 2).

### 3.3 Amino Acid Metabolism in Microglia

While little is known about how the reprogramming of amino acid metabolism affects the ability of microglia to respond to inflammation and mount an immune response, evidence shows that alterations in amino acid metabolism occur when microglia are activated (Table 2). For example, activated, proinflammatory microglia frequently exhibit increased glutamate synthesis, which, through the tricarboxylic acid cycle, can produce a significant amount of succinate. Elevated succinate has been shown to be a feature of proinflammatory microglia [113]. In contrast, Arg‐1 is frequently upregulated by anti‐inflammatory microglia; the precise function of this upregulation is not yet known, although it is generally linked to greater microglial phagocytosis, neuroprotective benefits, and improved tissue repair [114].

Altering the metabolism of amino acids can lead to modifications in the inflammatory phenotype and immunological response of microglia. On the one hand, the inflammatory response of microglia may be influenced by changes in important enzyme activities related to amino acid metabolism. One of the essential enzymes in controlling the conversion of arginine to succinate is aspartate aminotransferase (AAT), and blocking AAT activity has been shown to lower the generation of proinflammatory molecules, including NO and IL‐6, in microglia [91]. L‐arginine is converted to L‐citrulline by NOS, and NOS enzyme activity inhibition can reduce the level of hazardous NO produced by microglia [92]. On the other hand, glutamine can function as an alternative energy source for microglia and can regulate the immune system. Increasing glutamine intake by microglia can increase the metabolism of glutamine breakdown, increase ATP synthesis in microglia, and promote microglial phagocytosis of neutrophils, which reduces postischemic stroke neuroinflammation [89].

## 4 Perspectives

The immune response in the CNS relies heavily on microglia, which are promptly activated after an IS. Previously, activated microglia were believed to negatively impact stroke recovery by releasing different inflammatory cytokines. Controlling microglial activation to inhibit neuroinflammation has been widely recognized as a successful strategy for decreasing the damage caused by stroke. Despite the harmful impact of inflammation, microglia also provide different forms of neuroprotection following stroke, such as clearing cell waste and releasing growth factors. Additional investigations are needed to ascertain the effective application of these mechanisms in subsequent stroke therapies. It is also crucial to consider the polarization of microglia. Additional studies are therefore needed to explore the activation and polarization processes of microglia and to determine their potential applications in future stroke therapies. Microglia also engage with most cells in the CNS, overseeing their activity and generating various outcomes. Research into these interactions between pathways will increase our understanding of the pathological processes involved in stroke and other diseases of the CNS. The glycometabolism of microglia mainly includes OXPHOS and glycolysis, whereas lipid metabolism comprises lipid synthesis and lipolysis. Producing precursor substances for inflammatory agents may lead to the conversion of microglia into an anti‐inflammatory state. The metabolism of glucose, lipids, and amino acids is regulated by key enzymes, and their intermediate metabolites also affect cell phenotypic functions, thereby playing important roles in the poststroke inflammatory response and damage to neural function. Therefore, the study of microglial metabolic pathways and their products can provide new research ideas for the treatment of IS.

## Author Contributions

J.L. and Y.J. conceived this review and drafted the manuscript. J.L., X.Z., X.K., Y.C., L.L., and X.C. created the figures and collected and organized references. X.T. and D.D. reviewed and approved the submitted manuscript.

## Conflicts of Interest

The authors declare no conflicts of interest.

## Data Availability

The data that support the findings of this study are available from the corresponding author upon reasonable request.
